# GEM, a member of the GRAM domain family of proteins, is part of the ABA signaling pathway

**DOI:** 10.1038/srep22660

**Published:** 2016-03-04

**Authors:** Nuria Mauri, María Fernández-Marcos, Celina Costas, Bénédicte Desvoyes, Antonio Pichel, Elena Caro, Crisanto Gutierrez

**Affiliations:** 1 Centro de Biologia Molecular Severo Ochoa (CSIC – UAM), Nicolas Cabrera 1, Cantoblanco, 28049 Madrid, Spain

## Abstract

Abscisic acid (ABA) is fundamental for plant development. Multiple factors have been identified that participate in the ABA signaling network, although a role of many proteins still await to be demonstrated. Here we have investigated the role of GEM (GL2 EXPRESSION MODULATOR), originally annotated as an ABA-responsive protein. GEM contains a GRAM domain, a feature shared with other eight Arabidopsis proteins for which we propose the name of GRE (GEM-RELATED) proteins. We found that (i) *GEM* expression responds to ABA, (ii) its promoter contains ABRE sites required for ABA response, and (iii) *GEM* expression depends on members of the ABA signaling pathway. This is consistent with the expression pattern of *GEM* during development in plant locations were ABA is known to play a direct role. We also found that GEM binds various phospholipids, e.g. mono and diphosphates and phosphatidic acid, suggesting a potential link of GEM with membrane-associated processes. Consistent with this, we found that the phosphoinositol-4-phosphate kinase PIP5K9 binds GEM *in vivo*. Finally, we demonstrated a role of GEM in seed dormancy. Together, our data led us to propose that GEM is an ABA-responsive protein that may function downstream of ABI5 as part of the ABA signaling pathway.

Signaling pathways initiated by plant hormones are relevant for almost every aspect of plant physiology, including growth, development and response to stress. Plant hormones belong to two groups depending on their downstream cascade: whereas auxin, gibberellic acid and jasmonic acid are perceived by specific receptors and transmitted through selective proteolysis, abscisic acid, cytokinin and brassinosteroids are perceived by cytoplasmic and/or membrane-bound receptors and then a reversible protein kinase cascade transmits the signal to cellular factors that finally affect the expression of target genes^1^. Abscisic acid (ABA), in particular, is fundamental at several stages of plant development such as seed formation and dormancy, stomatal closure and response to a variety of stresses^2^. The ABA signaling pathway has been recently established as a core formed by the soluble ABA receptors PYRABACTIN RESISTANCE/PYRABACTIN RESISTANCE-LIKE/REGULATORY COMPONENT OF ABA RECEPTORS (PYR/PYL/RCARs), which inhibit the TYPE 2C PROTEIN PHOSPHATASES (PP2Cs). This in turn favors the accumulation of the active form of SNF1-RELATED PROTEIN KINASES (SnRK2s) that modulate the expression of a large variety of ABA-responsive genes^3^,^4^.

Three main families of transcription factors have been described in ABA response: the B3-, APETALA2- (AP2), and basic leucine zipper- (bZIP) domain families, represented by ABI3, ABI4 and ABI5, respectively^5^,^6^,^7^. Multiple ABA-responsive genes have been reported by transcriptomic assays. Many of them contain the G-box ABA Responsive Element (ABREs) binding sites in their promoter regions^8^,^9^. Other transcription factor binding sites are present in the regulatory regions of ABA-modulated genes^10^. In fact, ~10–15% of all genes are deregulated after drought or salt treatments in an ABA-dependent manner^9^,^11^,^12^. Therefore, the task of defining the functional relevance of putative ABA target genes is difficult due to their large amount and complexity.

The Arabidopsis genome contains 15 proteins that contain a GRAM (Glycosyltransferases, Rab-like GTPase Activators, Myotubularins) domain, 9 of which were also originally annotated as “ABA-responsive protein related” because of their homology with ABA45 in barley, which is induced by ABA^13^. The GRAM domain is found in a wide range of organisms and consists of a ~70 amino acids region^14^ folded as the pleckstrin homology domain^15^ and mostly studied in animal myotubularin proteins that bind to phosphoinositol^15^,^16^. Among the plant GRAM domain-containing proteins that lack the feature of being related to ABA response, several have been reported such as VAD1 (VASCULAR ASSOCIATED DEATH1)^17^, involved in defense response in vascular tissues, and MYOTUBULARIN1 (MTM1) and MTM2, which affect the dehydration stress-responding transcriptome^18^. Interestingly several members of the subgroup putatively related to ABA response have been identified independently by their interaction with other proteins such as FIP1 (FH INTERACTING PROTEIN 1)^19^, PRSL1 (PP1 REGULATORY SUBUNIT2-LIKE PROTEIN)^20^, and GEM (GL2 EXPRESSION MODULATOR)^21^. More recently GEM-RELATED5 (GER5) has been implicated in regulating seed development and inflorescence architecture based on its expression pattern, which was shown to be in part overlapping with that of GER1 and GEM in reproductive organs^22^. Furthermore, *ger5–1* mutants show reduced sensitivity to ABA and transcript changes in carbohydrate metabolism and catabolic processes^22^. The expression pattern of GRAM domain-containing proteins under various experimental settings, in particular those related to ABA, suggests that they may play functions in the perception of environmental signals and hormone signaling^23^. However, the potential relevance, if any, of GEM in ABA response has not been systematically explored.

Here, we focused on GEM to establish its role in ABA response by combining reporter gene expression studies and *GEM* expression studies in ABA signaling mutants with other molecular studies. We have found that *GEM* expression occurs in plant locations and stages associated with physiological increases of ABA. Accordingly, *GEM* is activated by ABA, in an ABRE-dependent manner, and requires some known regulators of the core signaling network of ABA. Germination assays showed that *gem-1* seeds had increased dormancy levels than wild type seeds. Together, our results led us to conclude that GEM is part of the ABA signaling pathway and acts as a negative regulator of germination.

## Results

### GEM belongs to a subfamily of the GRAM domain-containing proteins

The GRAM domain has been found in a wide range of organisms from prokaryotes to eukaryotes. In plants, >260 proteins have been identified to contain this domain, most of which are monodomain proteins, unlike in animals in which GRAM domain is combined with a wide variety of other domains^23^. In particular, the Arabidopsis genome encodes 15 of these proteins. Among them, five proteins contain, in addition to the GRAM domain, another domain such as the C2 domain related to Ca^2+^-mediated signaling processes^24^,^25^ or the myotubularin domain^18^. The remaining 10 proteins contain a single GRAM domain and include VAD1, involved in hypersensitive response^17^, which is unrelated to the other nine proteins (Supplemental Figure 1). Based on sequence homology and phylogenetic analysis the latter set of proteins form a subfamily formed by GEM and 8 members that we propose to name GRE (GEM-RELATED; GRE1 through GRE8; Supplemental Table 1). We use GRE because the acronym GER had been already taken for GERMIN1 (GER1; AT1G72610), GER2 (AT5G39190) and GER3 (AT5G20630)^26^, which are proteins unrelated to the GRAM domain family. The size of these 9 proteins is also an overarching characteristic that separated them, with 210–299 amino acids, from the others larger proteins, with 598–1027 amino acids. This characteristic may be important taking into account the involvement of this domain in cell signaling. It must be noted that two of these proteins had been identified by independent studies: FIP1 (FH INTERACTING PROTEIN 1)^19^ and PRSL1 (PP1 REGULATORY SUBUNIT2-LIKE)^20^, which would correspond to GRE1 and GRE3, respectively, according to the nomenclature proposed here. Also, while this work in progress, GER5/GRE5 has been studied in detail and shown to be involved in seed development and inflorescence architecture^22^.

**Figure 1:**
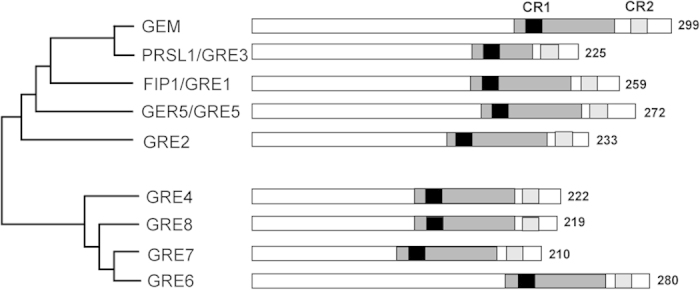
Amino acid homology relationship of GEM and GER proteins. GRE proteins are characterized by the GRAM domain (dark grey), that includes a highly conserved common region CR1 (black) and CR2 (pale grey) towards the C-terminus.

The GRAM domain of all GRE proteins is characterized by two unique motifs that differentiate them from the rest of GRAM domain proteins in *Arabidopsis*: (i) a highly conserved region we called CR1 (common region 1) with the amino acid motif CYL/ISTT/SxG (position 20–28 from the beginning of the GRAM domain; Fig. 1 and Supplemental Figure 2), and (ii) the common region 2 (CR2) with the amino motif DxxD/EFWFMGF, present close to the C-terminus (Fig. 1 and Supplemental Figure 2). Furthermore, we find other amino acids conserved throughout the sequence such as GGxE, just upstream of the GRAM domain (Supplemental Figure 2), and Y/HYR/K at the end of the GRAM domain, this latter lacking in GER3 sequence (Supplemental Figure 2). The role of these proteins is largely unknown, although their expression pattern under various experimental settings suggests that they may play functions in the perception of environmental signals and hormone signaling^23^. In this work, we focused on GEM to establish its potential role in this context.

### Expression pattern of GEM

To determine the spatial pattern of *GEM* expression we generated Arabidopsis transgenic plants (*pGEM*^*2774*^*::GUS*) expressing the *GUS* reporter gene under a 2774 bp sequence upstream of the ATG of GEM. Several independent lines were selected, showing a similar expression pattern, and the results described below are representative of them.

**Figure 2:**
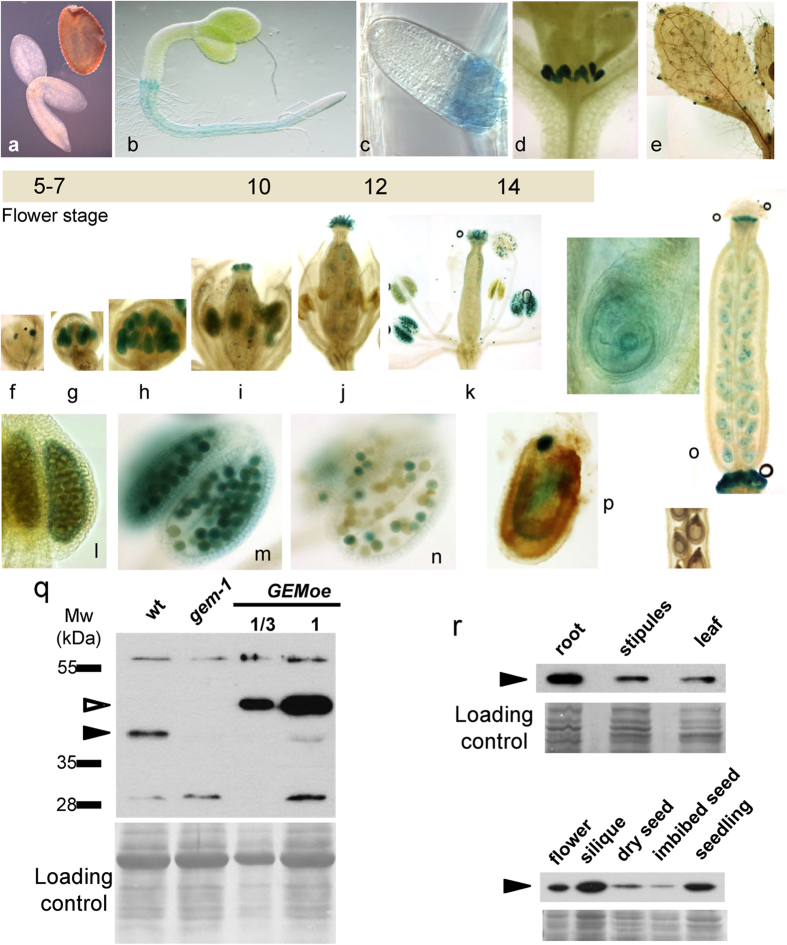
Spatial expression pattern of *GEM* at the gene (**a–p**) and protein (**q,r**) level, using GUS staining and Western blotting, respectively. The *pGEM*^*2774*^*::GUS* plants were used for this study. (**a,b**) young seedlings. (**c**) Emerging lateral root. (**d**) Stipules. (**e**) Hydathodes. (**f–k**) Details of flowers at different developmental stages. (**l-n**) Pollen grain development. (**o**) Developing silique. (**p**) Detail of a mature silique. (**q**) Rabbit antisera were raised using a GEM-specific N-terminal peptide and used to detect protein by Western blot in extracts of wild type, *gem-1* and *GEMoe* seedlings (14 day-old). Note that the band corresponding to GEM (black arrowhead) is not detectable in the *gem-1* mutant, whereas a strong band of HA-GEM (empty arrowhead) is detected in the *GEMoe* plants. Molecular weight markers are indicated at the left. (**r**) Detection of GEM protein (black arrowheads) in various organs, as indicated. Note that the label “stipules” refers to the shoot apex containing stipules.

This gene appears to be expressed throughout several stages of vegetative and reproductive development, including roots, several stages of gametogenesis and developing seeds, although with limited expression at different developmental times. After germination, GUS expression is not detected (or at very low levels) in young seedlings (2 days, Fig. 2a) and then was detected at higher levels in the roots, particularly at the transition and differentiated regions (5 days; Fig. 2b). The same GUS expression pattern was observed in the lateral roots (Fig. 2c). The aerial parts of the seedlings did not show significant GUS staining except in the stipules of 6–7 day-old seedlings (Fig. 2d). Leaves and other regions during vegetative development did not show GUS expression, except in the hydathodes (Fig. 2e).

At the reproductive stage (28 days), the *GEM* promoter is active at specific times of gametogenesis. A detailed analysis revealed a temporally limited expression pattern of GEM during pollen development (flower stage 8–10) (Fig. 2f–k). Thus, at anther stages I-IV, corresponding to pre-meiosis and meiosis, no expression was detected. At the post-meiotic stages V-VIII a clear expression was detected in anther primordia, including stages of microspore formation (Fig. 2f–h), although at later stages *GEM* expression is turned off. When the stamens have not yet grown over the ovary and pollination has not yet occurred *GEM* expression is also very apparent in the stigma (flower stage 12; Fig. 2i,j). A closer inspection revealed that *GEM* expression is particularly high at the tapetum layer (Fig. 2l). Then, the switch-off of *GEM* expression occurs concomitantly with the degradation of the tapetum layer. Expression decreases at later stages of pollen development (Fig. 2l–n). In the early stages of seed maturation (silique stages 3–5), expression is localized in all developing embryos within the embryo sacs, in places that may correspond to the endosperm layer (Fig. 2o). *GEM* expression is highly reduced at later stages when pigmentation of the seed coat becomes darker in the mature silique (Fig. 2p). This expression pattern is consistent with public microarray data (http://bbc.botany.utoronto.ca/efp/cgi-bin/efpWeb.cgi? primaryGene=AT2G22475&modeInput=Absolute).

To detect *GEM* expression at the protein level we generated rabbit polyclonal antibodies against a GEM-specific N-terminal peptide (see Methods). After purification of the polyclonal antisera with the peptide, we were able to detect GEM protein in whole cell extracts (Fig. 2q). A specific band was considered to be GEM since it was not detected in extracts of the *gem-1* mutant^21^, which carries out a T-DNA insertion in the second intron of the *GEM* gene. It is worth noting that the mobility of GEM protein under our conditions (apparent molecular mass ~37 kDa) was slightly lower than expected from its amino sequence (~32.2 kDa).

The abundance of *GEM* mRNA and protein correlate nicely as revealed by Western blot of extracts from different organs (Fig. 2r). At the vegetative stage, GEM protein is abundant in roots, but also detectable in samples of the shoot apex containing the stipules and in the leaves. In agreement with the *GUS* expression pattern, GEM was also detected in flower buds and in developing siliques but its level was lower in dry seeds and virtually absent in imbibed seeds (Fig. 2r).

### GEM expression is regulated by ABA

**Figure 3:**
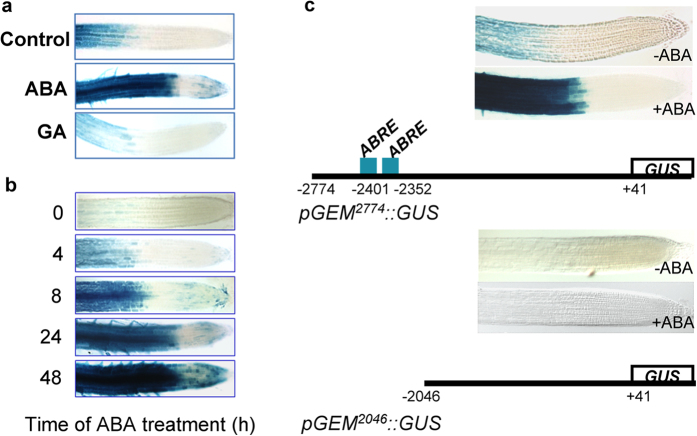
Response of *GEM* expression to ABA treatment. (**a**) GEM expression is activated 24 h after treatment with ABA (100 μM) but not with GA (25 μM). (**b**) Time-course of activation of *GEM* expression by ABA (100 μM). (**c**) Identification of ABA-responsive elements in the *GEM* promoter.

Previous studies suggested that GEM (and some GRE proteins) might be related to perception and/or signaling of hormonal response^13^,^23^. In fact, RNA *in situ* studies revealed that GEM, as well as GRE5/GER5 and GRE1/GER1, is expressed at the male and female reproductive organs^22^. We first used the *pGEM*^*2774*^*::GUS* plants to evaluate the transcriptional response of this gene to hormone treatments. We observed that the *GEM* promoter is strongly activated by ABA, but not by gibberellic acid (Fig. 3a). To evaluate the temporal pattern of *GEM* activation in response to ABA, we determined GUS activity at various times during ABA treatment and found that an increase in promoter activity was detected already 4 h after application of ABA (Fig. 3b).

To gain further insight into the mechanisms controlling GEM expression we analyzed its promoter. A detailed *in silico* search using the PlantPAN software^27^ predicted the presence of binding sites for various transcription factors. Among them, two consensus ABA responsive elements (ABRE) were identified at −2345/2352nt (ACGTGTC) and −2394/2401nt (ACGTGGC) upstream of the translation start site (Fig. 3c). Consistent with previous data, *pGEM*^*2774*^*::GUS* showed that *GEM* expression increases in response to ABA in the transition and differentiated regions of the root (Fig. 3c). Since the ABRE sites are relatively far from the ATG codon, which is not common to most of these elements in other ABA responsive genes^28^, we sought to determine whether they were required for ABA response by using plants expressing a shorter promoter lacking the putative ABRE elements (*pGEM*^*2046*^*::GUS*) (Fig. 3c). ABA treatment of these reporter plants did not result in a detectable stimulation of *GEM* expression under these conditions (Fig. 3c), indicating that the region containing the ABRE sites is required for full response to ABA treatment.

### *GEM* expression is activated by the ABA signaling pathway

**Figure 4:**
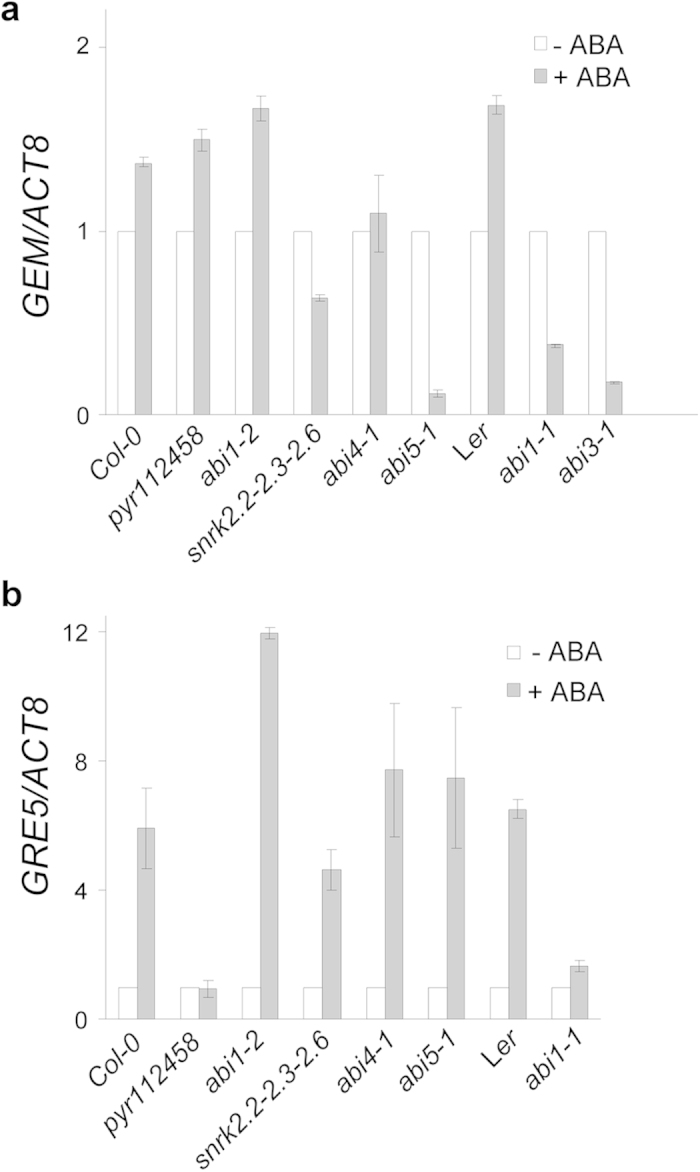
*GEM* and *GRE5/GER5* expression in ABA reposnsive mutant backgrounds. Relative amounts of (**a**) *GEM* and (**b**) *GER5/GRE5* transcripts in the indicated mutant backgrounds in the absence and the presence of a treatment with 10 μM ABA for 2 hours. Values from two independent biological replicates were normalized to the *ACT8* level and then to the value of GEM in each mutant. In all cases, except *abi4-1* in panel a and *pyr112458* and *abi1-1* in panel b, differences were statistically significant with at least p ≤ 0.05.

The signaling cascade controlling the response to ABA, although very complex, has been delineated to a considerable extent around a basic core formed by the PYR/PYL/RCAR receptors, the PP2C phosphatases and the SNRK Kinases^3^. To start defining how ABA controls *GEM* expression, we determined the mRNA levels of *GEM* by qPCR in a collection of mutants affected at different stages of the ABA response. We determined the response of *GEM* to a 2 h treatment with 100 μM ABA and found that *GEM* mRNA levels were not largely affected in *pyr112458*, *snrk2.2-2.3-2.6* and *abi4-1* mutants, whereas in the case of *abi1-2* and *abi1-1* we observed a small increase and decrease, respectively, in *GEM* levels. This behavior is consistent with the known ABA-related mutant phenotypes. In addition, GEM levels in *abi3-1* and *abi5-1* were significantly decreased, suggesting that GEM is a putative component in the ABA response pathway (Fig. 4a).

We also included the related *GER5/GRE5* gene in this analysis since it is the most responsive to ABA^29^. We found that *GER5/GRE5* mRNA levels in the various mutant backgrounds were different from those of GEM (Fig. 4b), which together with previous results^22^, reinforce the idea that they act at different stages in the ABA response.

### GEM binds phospholipids

**Figure 5:**
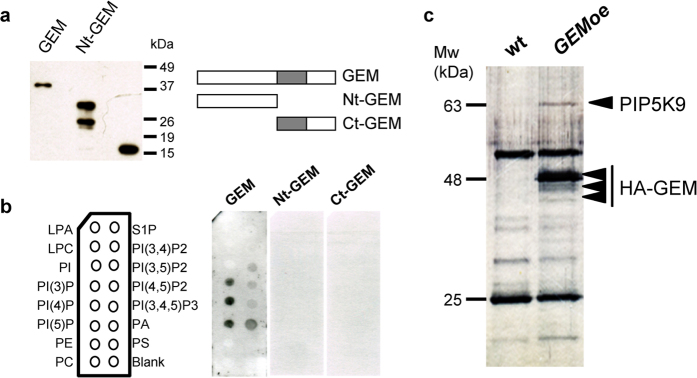
Identification of GEM-binding phospholipids and proteins. (**a**) Purification of bacterially-expressed full-length His-GEM proteins as well as its N-terminal and C-terminal moieties, as indicated. (**b**) Binding of polypeptides purified in (**a**) to various lipids was assessed by using membranes with lipids spotted as indicated in the left panel (LPA; lysophosphatidic acid; LPC, lysophosphocholine; PI, PtdIns; PI(3)P, PtdIns(3)phosphate; PI(4), PtdIns(4)phosphate; PI(5)P, PtdIns(5)phosphate; PE, phosphatidylethanolamine; PC, phosphatidylcholine; S1P, sphingosine-1-phosphate; PI(3,4)P2, PtdIns(3,4)diphosphate; PI(3,5)P2, PtdIns(3,5)diphosphate; PI(4,5)P2, PtdIns(4,5)diphosphate; PI(3,4,5)P3, PtdIns(3,4,5)triphosphate; PA, phosphatidic acid; PS, phosphatidylserine). Proteins bound were identified by Western blotting. (**c**) Identification of GEM-interacting proteins was carried by mass spectrometry. Extracts of wild type and *GEMoe* plants, expressing HA-GEM, were prepared and fractionated through polyacrylamide gel electrophoresis (a representative gel is shown here), as described in Methods. Proteins bands present uniquely in the *GEMoe* extracts were cut and subjected to mass spectrometry analysis. Molecular weight markers are shown at the left of the gel.

Animal GRAM domain-containing proteins have been reported to bind different phosphoinositides^30^,^31^,^32^. Plant proteins containing the pleckstrin homology (PH) domain, structurally related to the GRAM domain, bind phosphoinositides^18^,^33^. Therefore, we tested that possibility using GEM protein expressed in bacteria as a His-tagged protein as well as N- and C-terminal deletions (Fig. 5a) and a commercially available array of immobilized lipids. Full-length GEM protein binds strongly to PI3P, PI4P, PI5P and PA, and with less affinity to PI(3,5)P_2_, PI(4,5)P_2_ and PI(3,4,5)P_3_ (Fig. 5b). Neither the N-terminal moiety, nor the C-terminal that contains the GRAM domain, exhibited any detectable binding to these lipids, suggesting that binding requires the concerted action of several protein domains. It is worth noting that significant binding was detected only when the bacterially purified GEM protein was incubated with a plant extract, similar to previous reports^16^.

To gain further insight into the GEM functional network we sought to identify *in vivo* GEM protein interactors. To this end, we took advantage of the *GEMoe* plants^21^ that express a HA-tagged GEM protein. Soluble protein extracts were prepared from these plants as well as from wild type (Col-0) plants, and bound to agarose-antiHA beads. After washing, bound proteins were eluted, fractionated through polyacrylamide gels (Fig. 5c), and individual bands present only in the *GEMoe* sample were subjected to identification by mass spectrometry. First, we identified a doublet with a mobility of ~48 kDa as HA-GEM, confirming the adequacy of our purification protocol. In addition, a prominent *GEMoe*-specific band with an apparent mobility of ~63 kDa was identified as PIP5K9, a phosphoinositol-4-phosphate (PI4P) kinase that converts it into PI(4,5)P_2_. Although at this stage, the functional relevance of the GEM-PIP5K9 interaction remains to be addressed in the future, the finding serves to reinforce the idea that GEM might participate in lipid-mediated signaling processes. This may provide a link to the relevance of GEM for ABA response since PA is able to inhibit the function of ABI1^34^.

### Seed dormancy depends on correct levels of *GEM*

**Figure 6:**
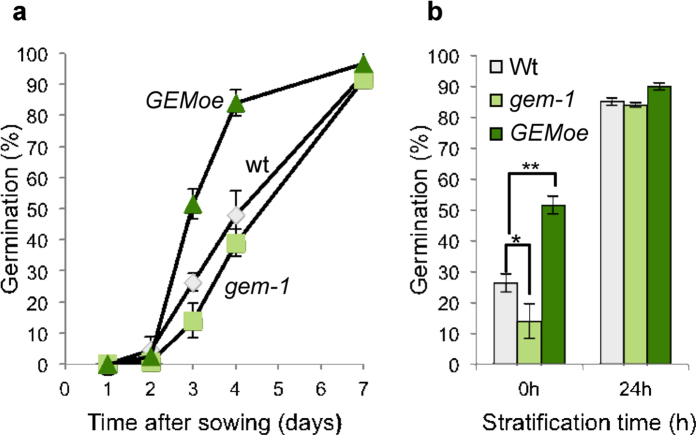
Relevance of GEM for dormancy and seed germination. (**a**) Germination frequency of wild type, *gem-1* and *GEMoe* seeds, as indicated, was estimated on a daily basis. (**b**) Effect of the stratification time on the seed germination phenotype of wild type, *gem-1* and *GEMoe* seeds, analyzed 3 days after sowing. Comparison with the wild type was assessed by the Student’s t test (*p ≤ 0.1; **p ≤ 0.05).

ABA is known to be part of the pathway controlling seed dormancy^3^,^35^. Based on our data relating GEM and ABA we sought to determine if GEM participates in seed dormancy. To this end, we plated freshly-collected wild type, *gem-1* and *GEMoe* seeds and quantified germination without stratification over a 7-day period when the wild type seeds showed a germination efficiency of ≥95% (Fig. 6a). The effect of GEM on seed germination was clearly observed by daily measurement of the germination efficiency in the absence of stratification. Under our conditions, the germination profile of *gem-1* seeds was delayed compared to the wild type seeds (clearly observed at 3 days after sowing) whereas the *GEMoe* seeds followed a slightly advanced profile (Fig. 6a). This effect was virtually lost after 24 h of cold treatment of the seeds (Fig. 6b).

## Discussion

A total of 260 proteins of 35 species have been identified within the *Viridiplantae* clade containing a GRAM domain. This is comparable to the amount of GRAM domain-containing proteins identified in Metazoan species^23^. In spite of the identification of these proteins, our knowledge on the function of plant GRAM domain-containing proteins is very scarce and only scattered studies have been reported on some of them^19^,^20^. Here, we have focused on GEM, a GRAM domain-containing protein previously identified by its ability to interact with the DNA replication protein CDT1 and modulate root hair patterning^21^. We have now studied GEM in the context of its putative ABA response function previously assigned by its similarity with ABA45^13^. Together with other related proteins they constitute a subfamily of 9 members, including GEM, that we propose to name GRE (for GEM-RELATED) and share a GRAM domain as their main identifiable structural feature.

The GRAM domain, as well as its structural homolog domain, PH domain, is thought to be an intracellular lipid-binding and protein-binding domain involved in membrane processes during cell signaling in animals^15^. In plants, GRAM domain proteins have been related with hormonal and environmental perception^14^, although a lipid-binding activity has not been demonstrated so far. GEM constitutes the first example since it binds strongly to phosphoinositide-monophosphate (PI(3)P, PI(4)P, PI(5)P) and PA (phosphatidic acid), and to a lesser extent to PI(3,4)P_2_, PI(4,5)P_2_, PI(3,4,5)P_3_. It is worth noting that these interactions are detected only when recombinant GEM protein expressed in bacteria is incubated with a plant extract, suggesting that some post-translational modifications and/or association with other plant proteins are required for efficient binding to phospholipids. The same requirement in a similar assay has been previously reported for animal GRAM domain-containing myotubularins^16^. Quite interestingly, in this work we have identified by mass spectrometry PIP5K9, a PI4P kinase that produces PI(4,5)P_2_, as a GEM-interacting protein. This protein-protein interaction has been found by *in vivo* immunoprecipitation experiments that typically reveal high affinity interactions. This finding directly relates GEM with phospholipid metabolism, as it is the case of other GRAM domain containing protein in Arabidopsis, e.g., MTM1^18^, which is involved in elevating the cellular level of PI(5)P in response to dehydration stress. Thus, PIP5K9 may be the protein that GEM requires for its phospholipid binding, a question that we relegate to future work. It is conceivable that GEM participates as a lipid transfer protein regulating the activity or location of signaling proteins as described about the GRAM domain of animal proteins^16^. In this sense, GEM, and also GRE3, has been recently reported to bind the serine/threonine phosphatase PP1c^20^, whose activity is regulated by PA binding^36^. In addition, root hair formation, where GEM has been implicated^21^, is regulated by membrane processes that implicate phospholipid kinases^37^ and phospholipid regulation^38^ linking lipid signaling with other GEM functions. The functional relevance of the GEM-PIP5K9 interaction remains to be addressed in the future. However, a PIP5K gene has been reported to be induced rapidly in response to drought, salt and ABA^39^. Together, our findings reinforces the relevance of GEM for ABA response, since PA is able to inhibit the function of ABI1^34^, and the idea that GEM might participate in lipid-mediated signaling processes.

ABA is known to control the expression of nearly ~10% of all Arabidopsis genes, of which more than half are activated^3^,^40^. The altered expression of certain ABA or stress response genes has been also described in mutants of GRAM domain-containing proteins^17^. Our data indicate that *GEM*, as well as *GER5/GRE5*, behaves as early response genes to ABA treatment, although with distinctive features. We have demonstrated that the region containing the two ABRE binding motifs, identified in ~20% of ABA-responsive genes^28^, are required for ABA-mediated *GEM* expression. Given the relatively long sequence upstream the GEM transcriptional start site we can not discard that regulation of GEM expression respond to several other transcription factors. We have observed that *GEM* mRNA levels do not increase in response to ABA treatment in the *abi5-1* mutant background. Interestingly, ABRE sequences are recognized by ABFs/AREBs, bZIP domain-containing transcription factors of which ABI5 is a member. This suggests that ABI5 might be a regulator of *GEM* expression in response to ABA. In further support of this conclusion, ABI5 is a target of SUMOylation by SIZ1^41^ and *GEM* expression in the *siz1* mutant does not respond to ABA treatment^42^. ABRE-mediated response to ABA appears to be a general feature of members of the GRE subfamily of GRAM domain proteins since, in addition to *GEM* and *GER5/GRE5*, the mRNAs of *GRE1*, *GRE4*, *GRE6*, *GRE7* and *GRE8* also increase after ABA treatment^43^. The promoters of all these genes, except *GRE7* and *GRE8*, contain at least one ABRE-like motif. The effect of ABA on *GEM* expression may serve to link, at least in part, two independent observations regarding the effect of GEM and of ABA on root hair patterning^21^,^44^. Moreover, the inhibitory activity of ABA on the expression of *CDT1a* could also add complexity to this network given the interaction of CDT1a and GEM^45^,^46^. Based on the ground work established here, the possible relevance of this interaction in ABA response could be an attractive topic for future research.

In addition to ABI5 and SIZ1, other factors appear to be required for full *GEM* expression, suggesting that it is finely controlled by a complex set of coordinated pathways. Thus, the use of plants carrying mutations in various genes involved in ABA signaling response led us to show that activation of *GEM* expression depends on well-known factors in the pathway. The *pyr1,pyl1,pyl2,pyl4,pyl5,pyl8* sextuple mutant in the receptors, the first element in the ABA signaling cascade^47^, has normal levels of *GEM* mRNA whereas those of *GER5/GRE5* are reduced, pointing to other members of the receptor family as responsible for activating *GEM* expression. The next step depends on the PP2C-type phosphatase ABI1, a negative regulator of the SnRK2 kinases^48^. We found that plants carrying the *abi1-1* allele, but not the *abi1-2* allele, do not increase *GEM* expression in response to ABA. This observation is consistent with the idea that *GEM* is a downstream target since (i) the *abi1-1* allele corresponds to a point mutation that abolishes binding to the PYR/PYL/RCAR receptors but not binding to the SnRK2 kinases, thus rendering a mutant ABI1 protein insensitive to ABA but maintaining its repressor activity on the SnRK2 kinases, and (ii) the *abi1-2* allele, on the contrary, is a null T-DNA insertion mutant that leads to a constitutively activated ABA signaling^49^,^50^. These data are in agreement with transcriptomic analysis of *abi1-1* and *abi1-2* mutant plants^29^. The SnRK2 kinases are positive regulators of ABA signaling and we have found that GEM expression in response to ABA, but not *GER5/GRE5* expression, is reduced in the *snrk2* triple mutant^51^. SnRK2 kinases phosphorylate various transcription factors involved in ABA signaling, including ABI5, a member of the bZIP AREB/ABF family, the B3 type domain ABI3 and the AP2/APETALA member ABI4^40^. As already mentioned, activation of *GEM* expression is abolished in the *abi5-1* mutant. Furthermore, a similar result was obtained in the *abi3-1* mutant, consistent with the participation of both ABI5 and ABI3 in *GEM* expression in response to ABA. Finally, the amount of *GEM* transcripts appears to be post-transcriptionally regulated by the 5′-3′-exoribonucleases XRN2 and XRN3, as deduced from the increased levels of *GEM* mRNA detected in the double *xrn2 xrn3* mutant^52^. Interestingly, XRN2 and XRN3 activity is inhibited in response to stress by lipid second messengers, which levels depend on the phospholipid phosphatase ALX8/FRY/SAL1. Consistent with a role in maintaining *GEM* transcript stability, the *alx8* mutant exhibits increased levels of *GEM* mRNA^53^, suggesting that *GEM* is a likely target of this post-transcriptional regulatory pathway. A different mechanism seems to apply to *GER5/GRE5* since its transcript level is not increased in the *alx8* mutant^53^.

The *GEM* expression pattern coincides temporally and spatially with the accumulation of ABA in several tissues and organs such as roots, the tapetum layer or endosperm during seed development^54^, the latter consistent with germination phenotype of *gem-1* plants. Endosperm accumulates lipid and protein storage and is the main tissue responsible for seed dormancy^55^,^56^. The slight, although clear, dormancy phenotype in *gem-1* plants is consistent with the hypothesis that *GEM* participates in ABA-mediated processes occurring during seed maturation. The increase in PA levels observed during seed development^57^ may be related to the ability of GEM to bind phospholipids. Thus, our results led us to tentatively place GEM as a positive effector of germination, necessary to break seed dormancy. However future studies are needed to unravel the complex role of GEM and the precise step of ABA signaling that is implicated.

## Methods

### Plant material and growth

*Arabidopsis thaliana* plants (Col-0 and L*er*) were grown on ½ MS salts medium and 1% sucrose plus 0.8% agar (16 h/8 h light/dark photoperiod), at 22˚C and 70% relative humidity. Plants older than 10d were grown on soil at the same conditions. The homozygous T-DNA insertion line *gem-1* (SALK_145846) and transgenic overexpressing line *GEMoe* have been described^21^. ABA-insensitive plants were provided by R. Solano (*abi1-2*), P.L. Rodríguez (*hab1-1 abi1-2*, *pyr1-pyl1-pyl2-pyl4-pyl5-pyl8, snrk2.2-snrk 2.3-snrk 2.6)* and O. Lorenzo (*abi1-1*, *abi3-1*, *abi4-1*, *abi5-1)*.

### Germination assays

For germination assays freshly harvested wild type Col-0, *gem-1* and *GEMoe* seeds were collected at the same time from plants grown in the same conditions. For each genotype, one hundred seeds were sown in triplicate, after 0–24 hours at 4 °C in darkness, on MS medium and incubated at 22 °C under 16 h/8 h light/dark conditions. Germination was scored every 24 hours by radicle emergence through the seed coat.

### Real-Time qPCR Analysis

Total RNA from seedlings and roots was extracted using Trizol reagent (Invitrogen). RT was carried out with the SuperScriptIII RT system (Invitrogen) using 500 ng of RNA as template and polydT primers. qPCR was performed in a LightCycler System using the FastStart DNA Master Green I (Roche) or an ABI Prism 7900HT SDS GoTaq qPCR master mix (Promega) with a 1:3–1:5 total cDNA dilution. The amount of actin (*ACT8*) mRNA was used for normalization before calculating fold changes. Data were generated from biological replicates. The following primer pairs were used to detect expression of: *ACT8* (AT1G49240), forward 5′AACGACCTTAATCTTCATGCTGC3′, reverse 5′GGTAACATTGTGCTCAGTGGTGG3′; *GEM* (AT2G22475), forward 5′AGATAGCCTTGTCCGATGAG3′, reverse 5′TCTCCATCGTATCTTTCAAC3′; *GER5/GRE5* (AT5G13200), forward 5′TCTGGCACAATCTGAAGACAGG3′, reverse 5′TGGTCAGCAGATGATGCGTAGC.

### Antibody generation and Western-Blot assays

Polyclonal antibodies (anti-GEMpep) were generated using a GEM N-terminal peptide (LSDEVEIETKGSDS) of the first exon (39–53 aa) as antigen to inoculate rabbits (Biogenes, Germany). After purification of the antisera using GEM-HA beads, the antibodies were validated and tested by Western blot analysis in extracts of total protein (50 mM Tris-HCl, pH 7.5, 150 mM NaCl, 5 mM MgCl_2_, 0.2% Nonidet P-40). After transfering to membranes and blocking with 5% milk, a 1:5000 anti-GEMpep dilution was used. GEM protein was detected using a chemiluminescent procedure (ECLplus western-blot detection system; Amersham Bioscience).

### GEM promoter:GUS fusions and transgenic plants

To generate the *pGEM*^*2774*^*::GUS* and *pGEM*^*2046*^*::GUS* constructs, the indicated fragments upstream of the translational start of *GEM* gene were amplified by PCR from the BAC F14M13 (GenBankAC006592) using primers 5′-GTCGACGCCAAGAAACCAAGAAAGAT-3′ paired with 5′-TTTAGGATCCACCTCAGTCTTCACTACG-3′, and 5′-TATAAAGCTTATACGAGCAAGGGCTG-3′ paired with 5′-TTTAGGATCCACCTCAGTCTTCACTACG-3′, respectively. These fragments, which also contained 41 bp of the N-terminal coding region of *GEM*, were cloned into pBI101 (Clontech) binary vector, fused in frame to the *GUS* gene, sequenced to verify the junctions of the chimeric constructs. Plants were transformed with the *Agrobacterium tumefaciens* strain C58CRifR using the floral dip method^58^. T_1_ seeds were plated on the germination medium containing 50 μg ml^−1^ of kanamycin.

### GUS assay

Histochemical GUS assays were performed as described^59^ with slight modifications. Plant material was infiltrated by vacuum with the GUS substrate (100 mM NaPO_4_, pH 7.0, 0.5 mM K_3_Fe(CN)_6_, 0.5 mM K_4_Fe(CN)_6_, 0,1% Tween-20 and 4 mM X-Gluc) for a maximum of 10 min, and incubated at 37 °C in darkness. Samples were cleared by several changes of 70% ethanol. Samples were examined with an Axioskop2 plus microscope (Zeiss) with digital Coolsnap FX camera (Roper Scientific).

### Binding to phospholipids

Constructs of *GEM* cDNA and its deletions *NtGEM* (coding for amino acids 1–170) and *CtGEM* (171–299) fused to 6xHis in a pRSET-B vector have been described^21^. Recombinant proteins were expressed in *E. coli* BL21 Rosetta after 3 hours of growth at 30 °C in liquid medium supplemented with isopropyl β-D-1-thiogalactopyranoside (IPTG) and purified using Ni-NTA beads (Qiagen). The phospholipid binding assay was done using PIP-Strips (P-6001, Echelon Biosciences, Salt Lake City, UT). The membrane was incubated with 0.5 μg of purified recombinant proteins, supplemented as indicated with 100 μg of total protein extracts of wild type plants. Immunoblot analysis was performed as described above using 1:3000 diluted mouse monoclonal anti-poly His antibody (Sigma, H1029) and 1:10000 anti-mouse antibody conjugated to horseradish peroxidase (Amersham).

### Identification of GEM-interacting proteins by mass spectrometry

To identify GEM-interacting proteins we performed a co-immunoprecipitation assay using extracts of HA-tagged *GEMoe* plants. Protein extracts were prepared from 1 g of 14 day-old *GEMoe* plants grown in liquid medium. Plants were ground and homogenized in 1 ml of extraction buffer (50 mM Tris-HCl, pH 7.5, 150 mM NaCl, 5 mM MgCl_2_, 0.2% Nonidet P-40) supplemented with plant protease inhibitor cocktail (Sigma) and phosphatase inhibitor cocktail (20 mM NaF, 0.1 mM Na orthovanadate, 5 mM Na pyrophosphate). The protein extract was sonicated on ice five times for 10 sec each and precleared with 50 μl of protein G-agarose for 15 min (10% flurry) (Santa Cruz Biotechnologies). After centrifugation at 3000 g, the cleared protein extract was incubated with protein G-agarose beads preincubated with anti-HA (Roche, 1:1000) with rotation for 1 h at 4 °C. Beads were washed five times with 1 ml of extraction buffer and finally proteins were eluted with 50 μl of 0.2 M glycine, pH 2.8 and immediately neutralized with 2 M Tris-HCl, pH 8.0. Proteins were fractionated by SDS-PAGE 8% long gels and transferred onto a nitrocellulose membrane. As a control, an extract of wild type plants (Col-0) was used. Protein bands detected by Coomassie Blue staining only in the *GEMoe* extract were extracted for peptide analysis. The molecular mass of tryptic fragments was measured with a MALDI Spectrometer and the sequence compared to the TAIR database.

## Additional Information

**How to cite this article**: Mauri, N. *et al*. GEM, a member of the GRAM domain family of proteins, is part of the ABA signaling pathway. *Sci. Rep*. **6**, 22660; doi: 10.1038/srep22660 (2016).

## Supplementary Material

Supplementary Information
