# Supplementary material for: GEM, a member of the GRAM domain family of proteins, is part of the ABA signaling pathway
Source: Sci Rep. 2016 Mar 4;6:22660. doi: 10.1038/srep22660 (PMC4778130; doi:10.1038/srep22660)
Supplement: Supplementary Information [file srep22660-s1.pdf]

Supplementary Information for

GEM, a member of the GRAM domain family of proteins, is part of the ABA signaling pathway

Nuria Mauri, María Fernandez-Marcos[1], Celina Costas[1,2], Bénédicte Desvoyes, Antonio Pichel, Elena Caro[3] and Crisanto Gutierrez*

Centro de Biologia Molecular Severo Ochoa (CSIC – UAM), Nicolas Cabrera 1, Cantoblanco, 28049 Madrid, Spain

[1] This should be considered shared co-authorship

[2] Current address: Universidad de Vigo, Vigo, Spain

[3] Current address: Centro de Biotecnologia y Genomica de Plantas, Madrid, Spain

* To whom correspondence should be addressed: cgutierrez@cbm.csic.es

Keywords: abscisic acid (ABA) – ABA signaling - GEM – GEM-related (GRE) – GRAM domains – phospholipid - germination – – *Arabidopsis thaliana*

Running title: GEM in the ABA signaling pathway

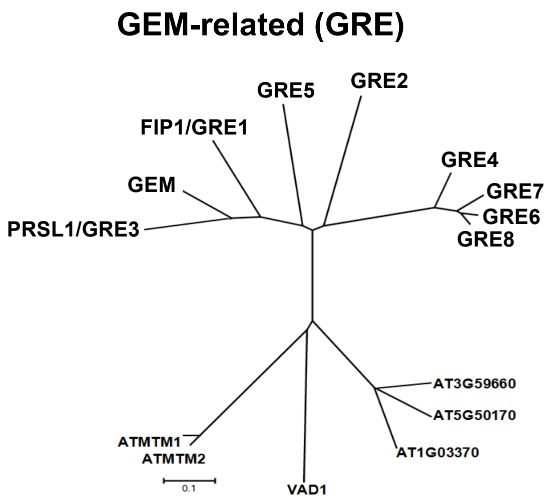

Supplemental Figure 1. Phylogenetic relationships of Arabidopsis GRAM domain-containing proteins. GEM and GRE proteins have been highlighted with a larger font.

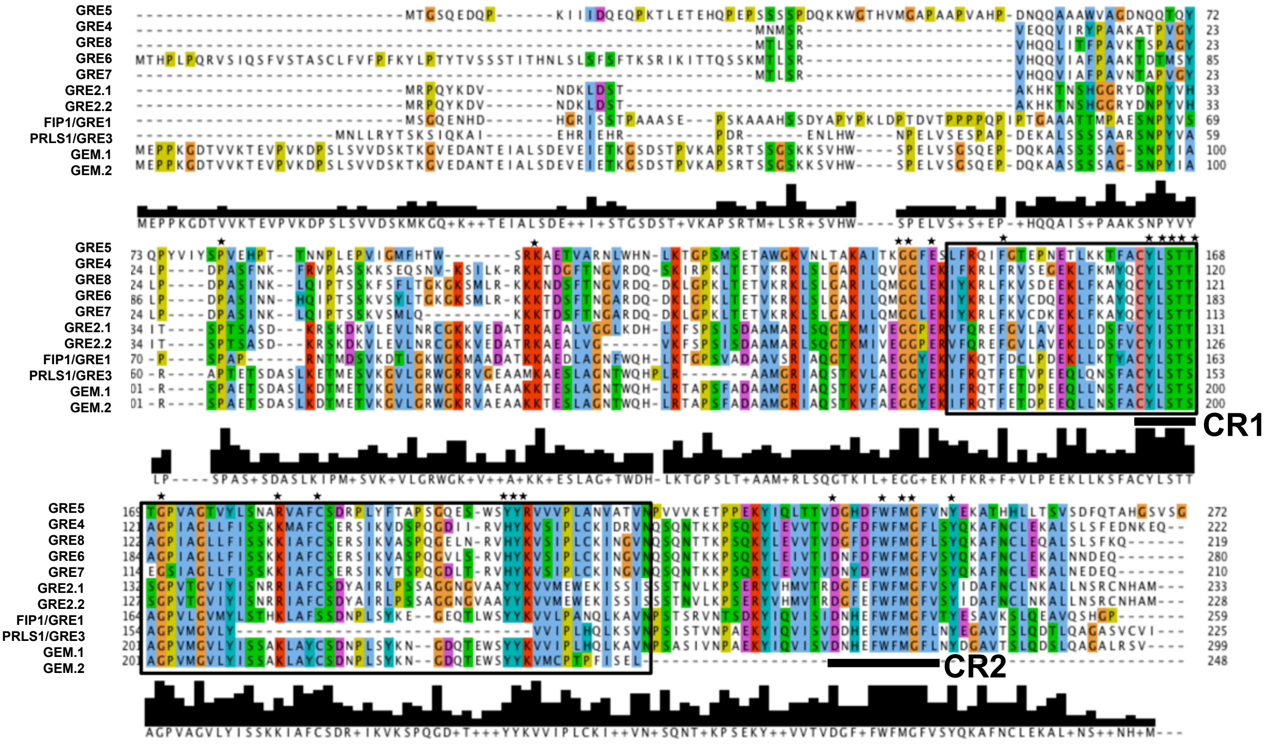

Supplemental Figure 2. Amino acid sequence homology of GEM and GRE proteins highlighting the GRAM domain (boxed) and the common regions CR1 and CR2. The alignment program used was MUSCLE (http://www.ebi.ac.uk/Tools/msa/muscle). Bar graph shows the relative homology of each amino acid position. Asterisks point to the amino acids conserved in all protein sequences analyzed.

Supplementary Table 1. GEM and GEM-related (GRE) proteins analyzed in this study.

AGI code	Name	Splice variant	Size (aa)	Mw (kDa)	GRAM (aa)
AT2G22475	*GEM*	.1	299	32.21	175-252 (75)
		.2	248	26.65	175-252 (75)
AT1G28200	*FIP1/GRE1*	.1	259	27.95	138-215 (77)
AT4G01600	*GRE2*	.1	233	25.91	106-185 (76)
		.2	228	24.34	106-185 (76)
AT4G40100	*PRLS1/GRE3*	.1	225	24.85	129-163 (34)
AT5G08350	*GRE4*	.1	222	25.25	95-173 (78)
AT5G13200	*GRE5*	.1	272	30.07	143-221(75)
AT5G23350	*GRE6*	.1	280	31.43	160-235 (75)
AT5G23360	*GRE7*	.1	210	23.64	88-166 (78)
AT5G23370	*GRE8*	.1	219	24.55	96-174 (75)
